# Associations between month/season of birth and health outcomes: an umbrella review of systematic reviews and meta-analyses

**DOI:** 10.7189/jogh.16.04234

**Published:** 2026-10-09

**Authors:** Zhuoya Zhang, Lin Wang, Yinghui Zhou, Jie Zhang, Cheng Tan

**Affiliations:** 1 Affiliated Hospital of Nanjing University of Chinese Medicine, Jiangsu Province Hospital of Chinese Medicine, Nanjing, China; 2 Yancheng TCM Hospital Affiliated to Nanjing University of Chinese Medicine, Yancheng, Jiangsu, China

**Keywords:** season of birth, month of birth, seasonality, health outcomes, umbrella review, systematic review, meta-analysis, evidence grading

## Abstract

**Background:**

The relationship between month or season of birth and long-term health outcomes remains an important but underexplored topic in public health. Evidence suggests that birth timing may influence disease susceptibility and broader health trajectories, reflecting interactions between biological and ecological factors. Clarifying these associations is essential for advancing prevention and a holistic view of health shaped by temporal exposures.

**Methods:**

We conducted an umbrella review of systematic reviews and meta-analyses indexed in PubMed, Embase, and Web of Science up to 2 July 2025. Eligible studies reported quantitative estimates for associations between birth month/season and objectively measured health outcomes. We used random-effects models to produce pooled estimates and evaluated heterogeneity, small-study effects, and bias. We graded associations using established class-of-evidence criteria and assessed methodological quality with AMSTAR 2.

**Results:**

We included 10 meta-analyses, comprising 614 unique associations (defined as individual pairwise analyses between a specific birth month/season exposure and a distinct health outcome). Of these, 45 associations were statistically significant, and 25 were eligible for evidence grading. No association met class I criteria, but one (January birth and schizophrenia) reached class II. The most consistent findings involved neurodevelopmental and psychiatric outcomes, with winter births linked to a modest increase in schizophrenia risk (odds ratio (OR) = 1.05) and spring births to multiple sclerosis. Most autoimmune, oncological, and perinatal associations were graded as weak (class III–IV) due to small effect sizes or heterogeneity.

**Conclusions:**

The strongest evidence for associations between month/season of birth and long-term health outcomes was restricted to specific neuropsychiatric outcomes, with most other associations graded as weak. Grading the credibility of these associations helps distinguish robust signals from exploratory findings, and provides a clear framework for future mechanistic and epidemiological investigations.

**Registration:**

PROSPERO: CRD420251110595

A wide range of epidemiological studies have reported associations between month or season of birth and long-term health outcomes, with growing evidence linking birth timing to a spectrum of physical and mental health conditions. For example, winter births have been linked to an elevated risk of schizophrenia (SZ) [1], while spring births are associated with a higher risk of multiple sclerosis [2]. Additionally, birth season correlates with cardiovascular mortality [3], birth weight, and pubertal timing; studies show that spring births are associated with higher birth weights and earlier pubertal onset [4]. A meta-analysis by Boland *et al.* also highlighted the role of maternal birth month temperature and rainfall in influencing fertility outcomes across regions [5], further extending these associations across generations.

Despite these findings, the research remains fragmented and inconsistent. For instance, Saatci [6] found that the association between birth month and SZ varied by region. Similarly, Vaiserman [7] noted that while birth season may impact longevity, the strength of these associations differs significantly across populations. Furthermore, Boland *et al.* [8] demonstrated how birth season-disease associations vary globally, influenced by regional factors and study design. These inconsistencies underscore the need for a systematic review to consolidate the evidence and clarify the relationship between birth season and long-term health outcomes.

Although several meta-analyses have explored the relationship between birth timing and specific diseases, none has systematically evaluated the credibility of these findings. Given the inherent uncertainties in observational research, umbrella reviews offer a robust framework for synthesising evidence from multiple studies and evaluating association strength, sample sizes, and potential biases [9,10]. This approach enables a comprehensive and transparent synthesis of complex topics like birth seasonality.

In this umbrella review, we aimed to fill this critical gap by systematically synthesising and grading the credibility of evidence on associations between birth month/season and a broad range of health outcomes across all published systematic reviews and meta-analyses. Specifically, we sought to distinguish robust, well-supported associations from weak or exploratory findings, and to provide a transparent evaluation of the methodological quality of existing research on this topic.

## METHODS

### Search strategy

We conducted and reported this umbrella review in accordance with the PRISMA 2020 statement guidelines [11]. We performed a comprehensive literature search across PubMed, Embase, and Web of Science from inception to 2 July 2025. The search strategy was developed with support from an experienced medical information specialist and included MeSH and free-text terms related to birth (*e.g.* ‘childbirth,’ ‘born’), timing (*e.g.* ‘month,’ ‘season’) and study type (*e.g.* ‘systematic review,’ ‘meta-analysis’). We applied no language restrictions during the literature search to minimise publication bias and ensure comprehensive capture of all eligible systematic reviews and meta-analyses on the topic (Table S1 in the **Online Supplementary Document**).

### Inclusion and exclusion criteria

We determined eligibility for inclusion using a ‘Population, Exposure, Comparison, Outcome, Study design’ framework [12]. Regarding population, we considered studies involving human participants as eligible, with no restrictions on age, gender, or geographic location. For exposure, we used birth month/season, classified using any validated method for determining birth timing or seasonality. For comparison, we included studies that compared different birth months or seasons. We did not require a specific season for inclusion, as long as the study included a clear comparison. For outcome, we considered studies investigating any health outcome – both physical and mental – as eligible, if the outcome was ascertained using objective measures, clinical diagnoses, or validated self-reports, where applicable. For the study design, we included only systematic reviews and meta-analyses of observational studies (cohort, case-control, or cross-sectional).

Exclusion criteria were as follows: lack of quantitative analysis (systematic reviews that did not incorporate a meta-analysis or failed to report quantitative effect sizes, confidence intervals, or other essential statistical data were excluded); non-relevant study designs (reviews based on animal studies, laboratory experiments, genetic polymorphisms, randomised controlled trials, or conference abstracts were excluded), minimum data requirement (only meta-analyses including at least three original studies were considered eligible, as this allows for the calculation of robust 95% prediction intervals (PIs) [9]).

When the number of studies was identical, we selected the review with the largest sample size [13]. When two or more reviews addressed the same research question, we prioritised the meta-analysis with the largest number of included primary studies [12]. We applied this rule to minimise overlap in primary studies across included meta-analyses during the study selection stage, thereby mitigating the risk of duplicated data bias in subsequent evidence synthesis.

To ensure consistency and reliability in study selection, four trained investigators (ZYZ, LW, JZ, and YHZ) independently screened the titles, abstracts, and full-text articles. The team resolved discrepancies through discussion and, when needed, consulted TC for final decisions.

### Data extraction

Four reviewers (ZYZ, LW, JZ, and YHZ) worked in pairs to independently extract key data from eligible studies using a predesigned extraction form. Extracted items included: first author and publication year, search databases and timeframe, quality assessment tool used for primary studies, number of included studies (with study design), number of participants or cases, health outcome(s), exposure category (*e.g.* spring *vs.* other seasons), and effect size with 95% confidence interval (CI). We resolved disagreements through discussion or consultation with a third reviewer (CT).

### Statistical analysis

We recalculated all meta-analyses using random-effects models, based on summary estimates extracted from the original meta-analyses. We performed analyses in Stata version 17.0 (StataCorp LLC, College Station, Texas, USA) and *R*, version 4.3.2 (R Foundation for Statistical Computing, Vienna, Austria). Pooled effect sizes with corresponding 95% CIs were estimated to quantify overall associations, and 95% PIs were calculated to project the range of effects in future studies [14]. We assessed between-study heterogeneity using the *I*^2^ statistic, which represents the proportion of total variance due to genuine differences rather than chance [15].

We evaluated small-study effects (SSE) using Egger’s test, which we applied only to meta-analyses with at least 10 primary studies [16]. For meta-analyses with fewer than 10 primary studies, we did not formally test small-study effects, and we note this limitation in the relevant results and limitations sections. We assessed excess significance bias (ESB) by comparing the observed *vs.* expected number of statistically significant results [17,18]. We also examined whether the largest contributing study yielded a nominally significant effect (*P* < 0.05), as a marker of robustness [14].

Finally, we graded each association according to established class of evidence (CE) criteria, categorising evidence strength into five levels (convincing, highly suggestive, suggestive, weak, nonsignificant) [9] (**Table 1**).

**Table 1:** Criteria used to classify the evidence

Classification and criteria
Convincing evidence (class I)
*>1000 cases*
*Significant summary associations (P < 1 × 10^−6^) per random-effects calculations*
*No evidence of small-study effects*
*No evidence of excess significance bias*
*Prediction intervals not including the null value*
*Largest study nominally significant (P < 0.05)*
*No large heterogeneity (I^2^<50%)*
Highly suggestive evidence (class II)
*>1000 cases*
*Significant summary associations (P < 1 × 10^−6^) per random-effects calculation*
*Largest study nominally significant (P < 0.05)*
Suggestive evidence (class III)
*>1000 cases*
*Significant summary associations (P < 1 × 10^−3^) per random-effects calculations*
Weak evidence (class IV)
*All other associations with P ≤ 0.05*
Nonsignificant association
*All associations with P > 0.05*

### Quality assessment

We evaluated the methodological quality of each included meta-analysis using the AMSTAR 2 checklist, a valid and reliable tool for appraising the methodological rigour of systematic reviews and meta-analyses [19]. This instrument consists of 16 items that assess both critical and non-critical domains. Each item is rated according to predefined criteria, and overall confidence in the review's results is classified as high, moderate, low, or critically low quality. Four trained investigators (ZYZ, LW, JZ, and YHZ) independently and in pairs applied the AMSTAR 2 checklist to assess the methodological quality of all eligible systematic reviews with meta-analyses. The authors resolved disagreements through discussion; when they could not reach consensus, a third reviewer made the final decision.

## RESULTS

### Study selection

We initially retrieved 4464 records from PubMed, Embase, and Web of Science. After deduplication, we screened 3087 articles against predefined eligibility criteria. We then excluded 3045 articles after reviewing titles and abstracts. Full-text assessments were then performed on the remaining 42 articles. Manual reference list checking of the included systematic reviews identified no additional eligible studies [20]. Ultimately, we included 10 systematic reviews with meta-analyses, representing 614 unique associations between birth month or season and health outcomes [1,21–29] (**Figure 1**; Tables S2 and S3 in the **Online Supplementary Document**).

**Figure 1:**
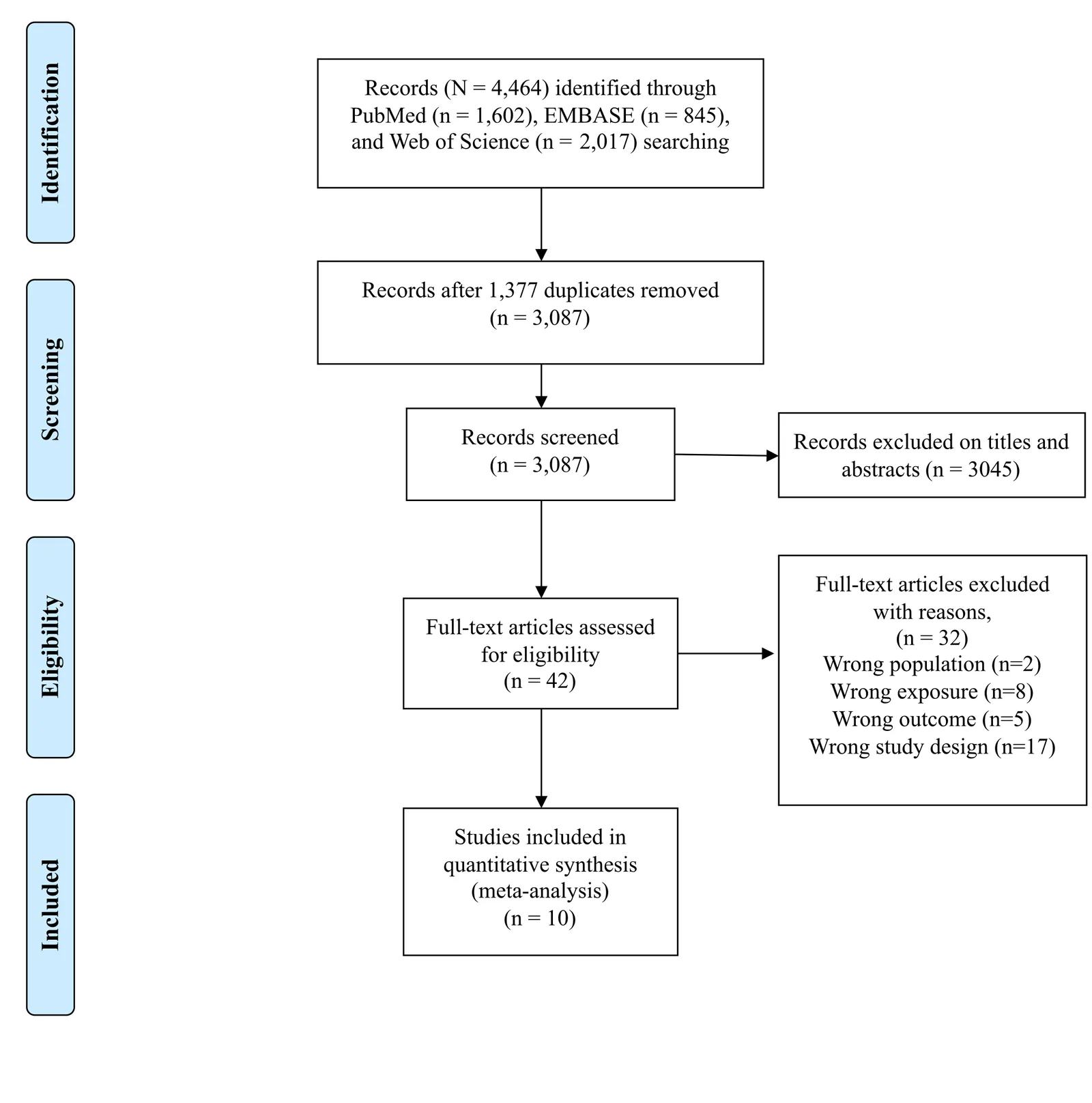
Study selection flow diagram.

### Overview of associations

A total of 614 unique associations were examined, encompassing a broad spectrum of health outcomes, including SZ, atopic dermatitis, bipolar disorder, major depressive disorder, alcohol use disorder, Alzheimer disease, anorexia nervosa, multiple sclerosis, and various cancers. Of these, 45 associations were statistically significant (*P* < 0.05). However, only 27 associations had the necessary data from the original studies. We classified these associations into four disease categories: autoimmune and allergic diseases, oncological diseases, neurodevelopmental and psychiatric disorders, and perinatal and maternal health (**Figure 2**).

**Figure 2:**
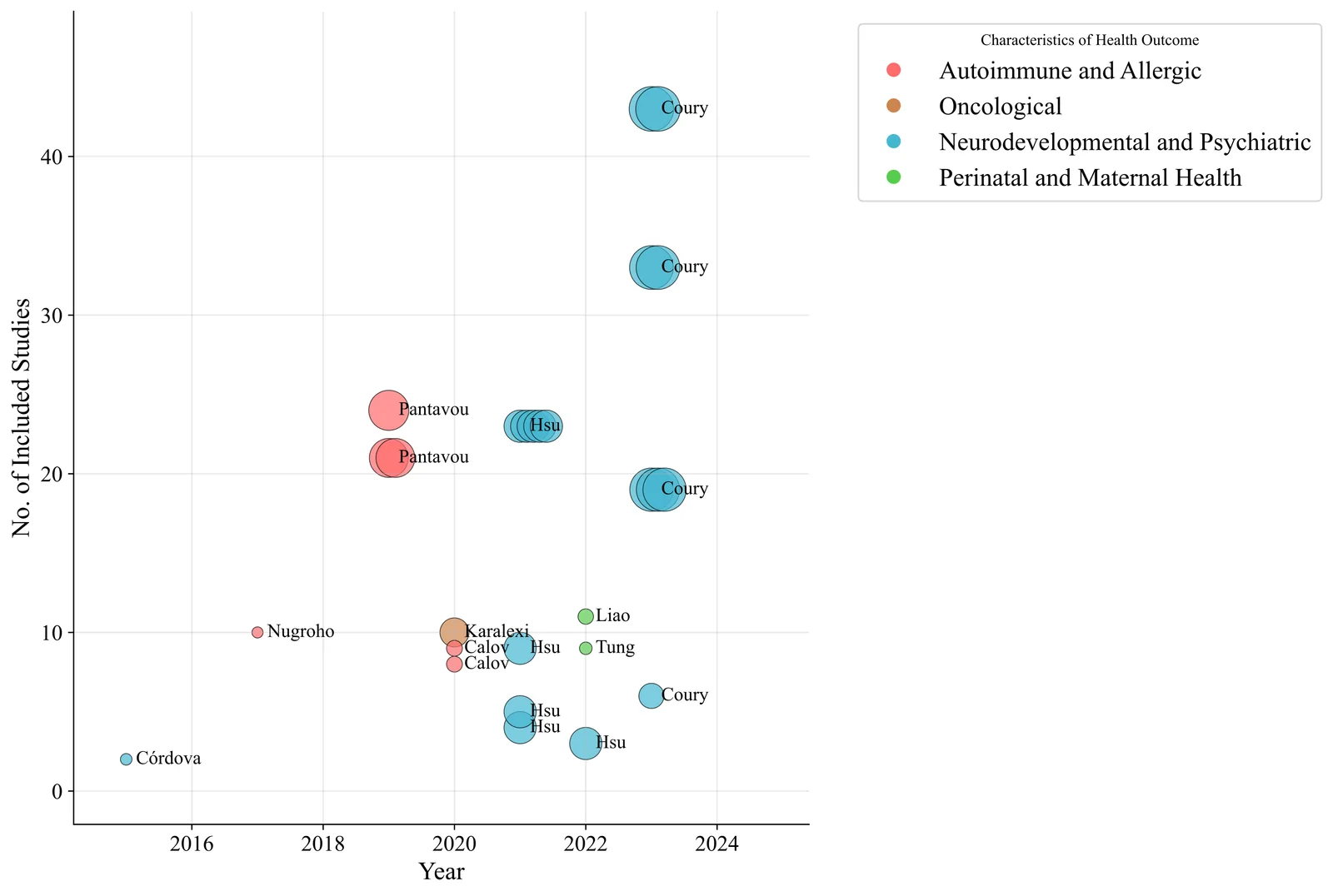
Characteristics of the included associations in the umbrella review. Each bubble represents an individual association and is colour-coded by health outcome category. Bubble size indicates total study population, and overlapping bubbles are offset horizontally to improve readability.

### Evidence grading

Evidence grading was based on two key aspects – the quality assessment of the included studies using AMSTAR and the CE evaluation for each association. All studies underwent AMSTAR quality assessments. However, only 25 associations with sufficient data were included in the final CE analysis, two fewer than shown in the bubble plot because these associations had incomplete data (**Table 2**; Table S4 in the **Online Supplementary Document**).

**Table 2:** Evidence from meta-analyses of associations between birth month/season and health outcomes

Source	Comparison	Health outcome	n	Random effects measures (95% CI)	Result	Cases, n	*P*-value	*I* ^2^	95% PI	SSE	ESB	LS	CE
Susanto *et al.*., 2017 [25]	Winter *vs.* summer	Level of cord blood IgE	10	1.27 (1.10–1.47)	Increased cord blood IgE level (>0.1 IU/ml)	1,834	1.47E-03	90.8	(0.75–2.15)	NO	NO	YES	IV
Pantavou *et al.*., 2020 [23]	Spring *vs.* non-spring	MS	24	1.08 (1.03–1.14)	Increased MS risk	146,537	2.22E-03	90.5	(0.87–1.35)	NO	NP	NO	IV
Pantavou *et al.*., 2020 [23]	April *vs.* non-April	MS	21	1.11 (1.05–1.18)	Increased MS risk	145,801	3.34E-04	83.1	(0.88–1.42)	NO	NP	NO	III
Pantavou *et al.*., 2020 [23]	November *vs.* non-November	MS	21	0.93 (0.89–0.97)	Decreased MS risk	145,801	5.62E-04	55.2	(0.82–1.06)	NO	NP	NO	III
Karalexi *et al.*, 2020 [26]	Winter *vs.* non-winter	Embryonal tumours	10	1.13 (1.01–1.27)	Increased embryonal tumour risk	1,463	3.51E-02	0.0	(0.99–1.30)	NO	NP	YES	IV
Calov *et al.*., 2020 [24]	Fall *vs.* spring	AD	9	1.16 (1.06–1.27)	Increased AD risk*	95,523	1.91E-03	59.5	(0.91–1.47)	NO	NP	YES	IV
Calov *et al.*., 2020 [24]	Winter *vs.* spring	AD	8	1.18 (1.05–1.33)	Increased AD risk	91,152	6.58E-03	66.9	(0.86–1.63)	NO	NP	YES	IV
Hsu *et al.*., 2021 [22]	January *vs.* non-January	SZ	23	1.05 (1.02–1.08)	Increased SZ risk	465,605	4.36E-07	43.3	(0.99–1.12)	NO	NO	YES	II
Hsu *et al.*., 2021 [22]	August *vs.* non-August	SZ	23	0.97 (0.94–0.99)	Decreased SZ risk	465,605	1.07E-02	56.6	(0.89–1.05)	NO	NP	NO	IV
Hsu *et al.*., 2021 [22]	September *vs.* non-September	SZ	23	0.97 (0.94–0.99)	Decreased SZ risk	465,605	1.27E-02	57.7	(0.89–1.05)	NO	NP	YES	IV
Hsu *et al.*., 2021 [22]	October *vs.* non-October	SZ	23	0.98 (0.96–0.99)	Decreased SZ risk	465,605	2.49E-04	0.0	(0.96–0.99)	NO	NP	NO	III
Hsu *et al.*., 2021 [22]	December *vs.* non-December	SZ	23	1.05 (1.01–1.08)	Increased SZ risk	465,605	4.53E-03	68.7	(0.94–1.16)	NO	NP	YES	IV
Hsu *et al.*., 2021 [22]	February *vs.* non-February	MDD	9	1.01 (1.004–1.03)	Increased MDD risk	751,702	6.05E-03	0.0	(1.00–1.03)	NO	NP	NO	IV
Hsu *et al.*., 2021 [22]	November *vs.* non-November	AUD	4	1.02 (1.0002–1.05)	Increased AUD risk	148,793	5.16E-02	0.0	(0.97–1.08)	NO	NO	YES	NS
Hsu *et al.*., 2021 [22]	September *vs.* non-September	Alzheimer disease	5	1.04 (1.001–1.07)	Increased Alzheimer disease risk	118,743	3.78E-02	15.7	(0.96–1.12)	NO	NP	NO	IV
Tung *et al.*., 2022 [29]	Fall *vs.* winter	PPD	9	0.86 (0.75–0.99)	Decreased maternal PPD risk	12,343	3.77E-02	67.1	(0.60–1.23)	NO	NP	NO	IV
Liao *et al.*., 2023 [28]	Winter *vs.* summer	Maternal HD	11	1.18 (1.02–1.38)	Increased maternal HD risk	16,402	2.75E-02	69.1	(0.75–1.87)	NO	NP	NO	IV
Coury *et al.*., 2023 [1]	Summer *vs.* non-summer	SZ	33	0.95 (0.93–0.98)	Decreased SZ risk (NH)	420,682	4.47E-05	72.2	(0.87–1.04)	NO	NO	YES	III
Coury *et al.*., 2023 [1]	Summer *vs.* non-summer	SZ	43	0.96 (0.94–0.98)	Decreased SZ risk (global)	440,039	2.92E-05	66.4	(0.87–1.04)	NO	NO	YES	III
Coury *et al.*., 2023 [1]	Winter *vs.* non-winter	SZ	33	1.05 (1.03–1.07)	Increased SZ risk (NH)	420,682	6.57E-07	30.9	(0.99–1.11)	NO	NO	NO	III
Coury *et al.*., 2023 [1]	Winter *vs.* non-winter	SZ	43	1.05 (1.03–1.07)	Increased SZ risk (global)	440,039	9.87E-07	26.8	(0.99–1.11)	NO	NO	NO	III
Coury *et al.*., 2023 [1]	January *vs.* non-January	SZ	19	1.05 (1.02–1.09)	Increased SZ risk (NH)	400,687	4.09E-04	64.1	(0.96–1.16)	NO	NP	YES	III
Coury *et al.*., 2023 [1]	August *vs.* non-August	SZ	19	0.94 (0.91–0.97)	Decreased SZ risk (NH)	400,687	5.48E-04	76.3	(0.84–1.06)	NO	NP	YES	III
Coury *et al.*., 2023 [1]	August *vs.* non-August	SZ	6	0.85(0.77,0.93)	Decreased SZ risk (SH)	7,491	6.04E-04	13.8	(0.71–1.02)	NO	NO	NO	III
Coury *et al.*., 2023 [1]	December *vs.* non-December	SZ	19	1.06 (1.02–1.10)	Increased SZ risk (NH)	400,687	9.44E-04	72.2	(0.94–1.20)	NO	NP	NO	III

AD – atopic dermatitis, AUD – alcohol use disorder, CE – class of evidence, CI – confidence interval, ESB – excess significance bias, HD – hypertensive disorders, LS – largest study, MDD – major depressive disorder, MS – multiple sclerosis, NH – Northern Hemisphere, NS – not significant, PI – prediction interval, PPD – postpartum depression, SH – Southern Hemisphere, SSE – small-study effects, SZ – schizophrenia, cb-IgE – cord blood immunoglobulin E

No evidence of small-study effects was detected *via* Egger’s test in the subset of meta-analyses with ≥10 primary studies eligible for this analysis. We observed no evidence of ESB across the included analyses. Further, 12 associations (48%) showed significant results from the largest study, reinforcing the consistency of the findings. 16 associations (64%) exhibited significant heterogeneity (*I*^2^ > 50%), indicating variability across studies. We classified evidence levels as follows: level II (one association), level III (nine associations), level IV (14 associations), and not significant (one association) (**Table 2**).

### Disease categories and findings

The 25 associations included in the final analysis were grouped into four categories (**Figure 3**), and a schematic diagram summarises the relationship between birth month/season and associated health outcomes

**Figure 3:**
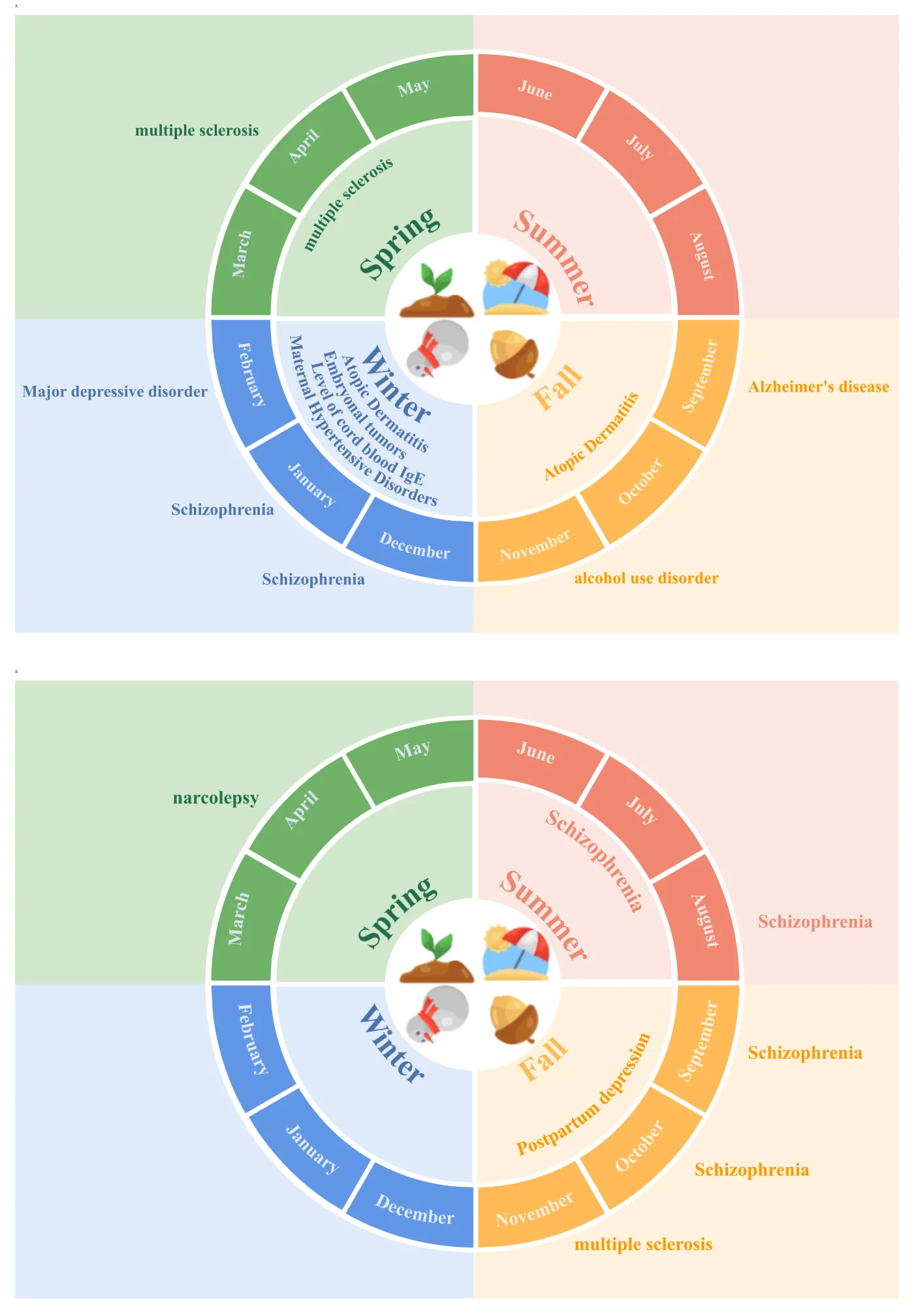
Summary of statistically significant associations between birth month/season and health outcomes identified in this umbrella review. **Panel A.** Health outcomes with increased effect sizes associated with birth month/season. **Panel B.** Health outcomes with decreased effect sizes associated with birth month/season. Curved text in the inner ring indicates season-disease links and horizontal text in the outer ring indicates month-disease links.

### Neurodevelopmental and psychiatric disorders

The most significant associations were observed in the neurodevelopmental and psychiatric disorders category, where several conditions showed distinct seasonal patterns.

Furthermore, SZ exhibited the strongest association, with winter births linked to an elevated risk (odds ratio (OR) = 1.05; 95% CI = 1.03–1.07). Specifically, January and December births were associated with a significant increase in SZ risk (OR = 1.05; 95% CI = 1.02–1.08 for January and OR = 1.05; 95% CI = 1.01–1.08 for December). In contrast, August births showed a reduced risk (OR = 0.97; 95% CI = 0.94–0.99). These associations were classified as level II–IV evidence, reflecting weak to moderate evidence with large sample sizes and consistent findings across multiple regions.

For major depressive disorder, a slight increase in risk was found for individuals born in February (OR = 1.01; 95% CI = 1.004–1.03). This association was classified as level IV evidence, indicating weak support due to small effect sizes and variability across studies.

Alcohol use disorder showed a marginal increase in risk for individuals born in November (OR = 1.02; 95% CI = 1.0002–1.05). However, this association was not significant, likely because the analysis tool's precision was limited when calculating values to the fourth decimal place.

Similarly, Alzheimer disease was slightly more common in those born in September (OR = 1.04; 95% CI = 1.001–1.07), also classified as level IV evidence.

### Autoimmune and allergic diseases

Following neurodevelopmental and psychiatric disorders, the autoimmune and allergic diseases category also revealed clear seasonal associations.

In multiple sclerosis, spring birth was linked to an elevated risk (OR = 1.08; 95% CI = 1.03–1.14; n = 146,537), with April birth further increasing the risk (OR = 1.11; 95% CI = 1.05–1.18). Conversely, November birth was associated with a reduced risk of MS (OR = 0.93; 95% CI = 0.89–0.97). The overall evidence was classified as level III, suggesting moderate to strong confidence due to large sample sizes but some heterogeneity across studies.

For cord blood immunoglobulin E (IgE) levels, winter birth was associated with a higher risk of elevated cord blood IgE (>0.1 IU/ml) with an OR of 1.27 (95% CI = 1.10–1.47). This was classified as level IV evidence, reflecting moderate to weak confidence due to study variability.

In atopic dermatitis, similar trends were observed. Both fall birth (OR = 1.16; 95% CI = 1.06–1.27) and winter birth (OR = 1.18; 95% CI = 1.05–1.33) were associated with a higher risk of atopic dermatitis compared to spring birth. This was classified as level IV evidence, reflecting weak support, a moderate sample size, and variability. However, when considered together with cord blood IgE levels, both conditions seem to reinforce each other’s findings.

### Perinatal and maternal health

The perinatal and maternal health category revealed important seasonal associations with postpartum depression (PPD) and maternal hypertensive disorders.

Although this section focuses on maternal health, we intended to examine results by birth month/season; therefore, we did not exclude the influence of the infant’s birth timing on the mother’s health from the analysis.

Fall delivery was associated with a lower risk of maternal PPD than winter delivery (OR = 0.86; 95% CI = 0.75–0.99; n = 12,343 cases), which we classified as level IV evidence because of weak confidence in the association.

For maternal hypertensive disorders, winter delivery compared with summer delivery was linked to a modestly higher risk (OR = 1.18; 95% CI = 1.02–1.38; n = 16,402 cases), which we also categorised as level IV evidence, offering a new perspective on cardiovascular diseases.

### Oncological diseases

In the oncological diseases category, the most significant finding involved embryonal tumours. Winter birth was associated with an increased risk of embryonal tumours (OR = 1.13; 95% CI = 1.01–1.27; n = 1463 cases), but the evidence was classified as level IV due to small sample sizes and lower significance.

## DISCUSSION

This umbrella review offers the first graded synthesis of evidence linking birth month and season to long-term health outcomes. Although many associations have been reported, the more strongly supported evidence is largely confined to neuropsychiatric outcomes, particularly schizophrenia, with weaker findings across autoimmune, oncological, and perinatal domains.

Potential mechanisms underlying these associations should be interpreted cautiously. Birth month and season are better regarded as ecological proxies for time-varying environmental and biological conditions than as direct exposures. Given the modest effect sizes observed, such interpretations remain hypothesis-generating rather than causal.

### Biological considerations relevant to the observed associations

Birth month/season may reflect seasonally varying environmental and biological conditions, including UV radiation (UVR), temperature, infection prevalence, circadian rhythms, air quality, and nutrition, among other factors that fluctuate with the seasons and months. Among these, UVR and temperature are widely used indicators of seasonality in epidemiological research. The following sections focus primarily on UVR and temperature as representative seasonal factors for discussing possible mechanisms, while also acknowledging that other seasonally varying influences may contribute to the observed associations.

### UVR-related considerations

UVR is one of the most frequently discussed seasonal factors in relation to birth timing and later health outcomes. Several lines of evidence may be relevant.

First, UVR may influence immune regulation through skin-mediated pathways. Experimental and translational studies have shown that UVR can trigger keratinocyte-derived inflammatory signalling [30–38], including the release of damage-associated molecular patterns and downstream activation of innate immune responses.

Second, UVR has long been discussed in relation to vitamin D synthesis, particularly in the context of immune-mediated and neuropsychiatric outcomes such as multiple sclerosis [39-41]. Reduced UV B (UVB) exposure during sensitive periods may influence vitamin D-related pathways, although this remains an indirect explanatory framework rather than a demonstrated causal mechanism in the present review.

Third, seasonal variation in UVR may correlate with differences in infection exposure or host susceptibility, providing an additional indirect context for some season-of-birth associations [42–44]. Other photobiological pathways, including α-melanocyte-stimulating hormone/melanocortin 1 receptor-related signalling, have also been proposed, although their relevance to the associations identified here remains uncertain [45–48]. Taken together, UVR-related evidence offers biologically plausible context for some of the observed patterns.

### Temperature-related considerations

Ambient temperature is another major seasonal correlate that may help contextualise associations with birth timing. Seasonal and monthly fluctuations in temperature have been associated with adverse pregnancy outcomes, maternal physiological stress, and altered foetal developmental conditions [49]. Temperature-related effects have also been discussed in relation to foetal brain development and later neuropsychiatric risk, including schizophrenia, although these pathways remain difficult to isolate from the broader seasonal environment [50]. In addition, maternal mental health may be sensitive to thermal stress; for example, higher ambient temperature during the perinatal period has been associated with increased postpartum depressive symptoms in a large population-based study from Taiwan [51]. These observations suggest that temperature may represent one relevant component of the broader seasonal context captured by birth timing, but it should not be interpreted as a singular explanatory exposure.

### Other seasonally varying factors

In addition to UVR and temperature, other seasonally varying influences may also be relevant to the associations examined in this literature. These include circadian rhythms, air pollution, and dietary variation, all of which may differ substantially across geographic, climatic, and social contexts. Maternal circadian disruption has been linked to altered metabolic regulation in offspring [52], while prenatal exposure to ambient air pollution has been associated with adverse birth outcomes through inflammatory, oxidative, and placental mechanisms [53–56]. Seasonal variation in maternal diet and nutritional status has likewise been proposed to contribute to developmental differences relevant to later health [57–59]. However, these influences are unlikely to operate uniformly across outcomes or populations, and their relevance cannot be inferred directly from birth month/season alone. They are therefore best regarded as examples of the broader seasonal context for which birth timing may serve as a proxy marker.

### Limitations

Several limitations should be considered when interpreting this review’s findings. First, birth month/season is a proxy variable rather than a direct exposure, and likely reflects a composite of environmental, biological, and social influences that vary across settings. Second, season definitions and climatic context differ across hemispheres, but hemisphere-specific reporting was not consistently available across the included meta-analyses, limiting further stratified analysis. Third, the overall strength of evidence remained limited: no association met Class I criteria, most associations were graded as weak, and even the strongest reported associations were modest in magnitude. Their statistical significance may partly reflect the very large sample sizes of the included meta-analyses rather than clinically meaningful individual-level effects.

Another key limitation relates to the quantification of primary study overlap across included meta-analyses. The corrected covered area (CCA) [60,61] index is the validated gold-standard method for formal overlap assessment in umbrella reviews; however, this study could not reliably calculate CCA because most included meta-analyses did not report unique identifiers for their underlying primary studies. While our a priori review selection rule likely reduced the risk of overlap, some residual unquantified overlap may still have affected the precision of the pooled estimates and evidence grading.

### Future research directions

Future research should move beyond using birth month/season as a proxy marker and instead seek to characterise the underlying seasonal conditions that birth timing may proxy. Integrating more granular environmental data with longitudinal birth cohorts, along with multi-omics approaches such as epigenomics, metabolomics, and microbiome profiling, may help clarify whether seasonally varying early-life contexts leave measurable biological signatures. In particular, prospective studies and individual participant data meta-analyses with trimester-specific exposure assessment, genetic information, and detailed geographic and climatic data may improve the resolution of future analyses and help identify potentially relevant windows of susceptibility.

Given the heterogeneity of outcomes and the generally modest magnitude of the associations observed in this review, future work should prioritise direct measurement of putative seasonal correlates – such as photoperiod, UV radiation, ambient temperature, infection patterns, maternal circadian disruption, air pollution, and nutritional variation – rather than relying on birth timing alone. Broader representation of tropical, urban, and resource-limited settings will also be important, as seasonal patterns and their biological correlates may differ substantially across contexts. In addition, ongoing environmental change, including climate change and artificial light exposure, may alter traditional seasonal patterns and should be considered in future studies. Overall, future research should aim to determine which specific seasonal exposures, if any, underlie the statistical patterns observed in relation to birth month/season.

## CONCLUSIONS

We highlighted associations between birth month/season and certain health outcomes, emphasising the need for further exploration. Highly suggestive evidence was found for neuropsychiatric conditions, particularly schizophrenia, but many associations remain weak. Future research is needed to clarify these associations and explore underlying seasonal correlates and potential mechanisms through broader, higher-quality studies.

## Additional material


Online Supplementary Document

## Data Availability

**Data availability:** This umbrella review generated no primary data. The data supporting the findings are available within the article and the **Online Supplementary Document**.
