# Supplementary material for: Associations between month/season of birth and health outcomes: an umbrella review of systematic reviews and meta-analyses
Source: J Glob Health. 2026 Oct 9;16:04234. doi: 10.7189/jogh.16.04234 (PMC13650975; doi:10.7189/jogh.16.04234)
Supplement: Supplementary file 1 [file jogh-16-04234-s001.pdf]

Supplement to: Zhang Z, Wang L, Zhou Y, Zhang J, Tan C. Associations between month/season of birth and health outcomes: an umbrella review of systematic reviews and meta-analyses. J Glob Health. 2026;16:04234.

Table S1. Search strategy from database inception to 2 July 2025

PubMed		
Search	Query	Results
#1	"birth s"[All Fields] OR "birthed"[All Fields] OR "birthing"[All Fields] OR "parturition"[MeSH Terms] OR "parturition"[All Fields] OR "birth"[All Fields] OR "births"[All Fields] OR "parturition"[MeSH Terms] OR "parturition"[All Fields] OR "born"[All Fields] OR "parturition"[MeSH Terms] OR "parturition"[All Fields] OR "childbirth"[All Fields] OR "childbirths"[All Fields]	619,250
#2	"season s"[All Fields] OR "seasonability"[All Fields] OR "seasonable"[All Fields] OR "seasonably"[All Fields] OR "seasonal"[All Fields] OR "seasonalities"[All Fields] OR "seasonality"[All Fields] OR "seasonally"[All Fields] OR "seasonals"[All Fields] OR "seasons"[MeSH Terms] OR "seasons"[All Fields] OR "season"[All Fields] OR "season s"[All Fields] OR "seasonability"[All Fields] OR "seasonable"[All Fields] OR "seasonably"[All Fields] OR "seasonal"[All Fields] OR "seasonalities"[All Fields] OR "seasonality"[All Fields] OR "seasonally"[All Fields] OR "seasonals"[All Fields] OR "seasons"[MeSH Terms] OR "seasons"[All Fields] OR "season"[All Fields] OR "season s"[All Fields] OR "seasonability"[All Fields] OR "seasonable"[All Fields] OR "seasonably"[All Fields] OR "seasonal"[All Fields] OR "seasonalities"[All Fields] OR "seasonality"[All Fields] OR "seasonally"[All Fields] OR "seasonals"[All Fields] OR "seasons"[MeSH Terms] OR "seasons"[All Fields] OR "season"[All Fields] OR "month"[All Fields] OR "months"[All Fields] OR "monthly"[All Fields]	2,442,682
#3	"systematic review"[Publication Type] OR "systematic reviews as topic"[MeSH Terms] OR "systematic review"[All Fields] OR "meta analysis"[Publication Type] OR "meta analysis as topic"[MeSH Terms] OR "meta analysis"[All Fields]	547,085
#4	#1 AND #2 AND #3	1,602
**EMBASE**		
Search	Query	Results
#1	birth OR born OR childbirth	818419
#2	seasonal OR season OR seasonality OR month OR monthly	1476517
#3	systematic AND review OR 'meta analysis'	948366
#4	#1 AND #2 AND #3	845
**Web of Science**		
Search	Query	Results
#1	TS=(Birth OR born OR childbirth)	1,098,024
#2	TS=(Seasonal OR Season OR Seasonality OR month OR monthly)	2,354,540
#3	TS=(systematic review OR meta-analysis)	876
#4	#1 AND #2 AND #3	2,017

Table S2. Characteristics of included studies

Author, year	Search databases (time)	Quality for primary studies	No. of studies (major design)	Participants/ Cases	Health outcome	Exposure (comparison)	ES (95%CI)
Coury et al., 2023	PubMed, Ovid, and Web of Science (June 22, 2020)	NR (NOS)	43 (Cohort and case-control studies)	14177395/ 440039	Schizophrenia	Season of birth:	OR (NH vs. SH vs. Overall):
						Spring vs. non-spring	1.01 (0.99–1.03) vs.0.99 (0.92–1.06) vs.1.00 (0.98–1.03)
						Summer vs. non-summer	**0.95 (0.93–0.98)** vs. 0.96 (0.92–1.01) vs.**0.96 (0.94–0.98)**
						Fall vs. non-fall	0.98 (0.95–1.01) vs.1.00 (0.95–1.04) vs.0.98 (0.96–1.00)
						Winter vs. non-winter	**1.05 (1.03–1.07)** vs.1.01 (0.96–1.07) vs.**1.05 (1.03-1.07)**
						Month of birth:	OR(NH vs. SH):
						Jan. vs. non-Jan.	**1.05(1.02-1.08)** vs.1.01 (0.87-1.18)
						Feb. vs. non-Feb.	1.03(0.99-1.08) vs.0.88(0.80-0.98)
						Mar. vs. non-Mar.	1.00(0.95,1.04) vs.0.97(0.86,1.11)
						Apr. vs. non-Apr.	1.00(0.97,1.03) vs.0.87(0.77,0.99)
						May vs. non-May	1.00(0.97,1.03) vs.0.97(0.86,1.10)
						Jun. vs. non-Jun.	0.98(0.95,1.01) vs.1.11(0.95,1.29)
						Jul. vs. non-Jul.	0.98(0.95,1.00) vs.0.95(0.85,1.06)
						Aug. vs. non-Aug.	**0.94(0.91,0.97)** vs.**0.85(0.78-0.93)**
						Sep. vs. non-Sep.	0.97(0.94,1.00) vs.1.04(0.85,1.26)
						Oct. vs. non-Oct.	0.98(0.97,1.00) vs.1.01(0.92,1.11)
						Nov. vs. non-Nov.	1.01(0.99,1.03) vs.0.95(0.85,1.06)
						Dec. vs. non-Dec.	**1.06(1.02-1.10)** vs.1.13(0.89-1.43)
Calov et al., 2020	PubMed, Embase, and Web of Science (April 21, 2018)	7 high-quality research (NOS)	9 (cross-sectional)	726,378/ 93,890	Atopic Dermatitis	Season of birth:	OR
						Summer vs. Spring	1.04(0.95-1.13)
						Fall vs. Spring	**1.16(1.06-1.28)**
						Winter vs. Spring	**1.15(1.04-1.27)**
Hsu et al., 2021	PubMed, Embase, and Cochrane (December 31,2020)	No high bias research(Hoy's risk of bias tool)	51(Observational studies)	>29074024/ 465605	Schizophrenia	season of birth:	RR
						Spring vs. non-spring	1.002（0.984-1.021）
						Summer vs. non-summer	0.974（0.960-0.989）

Author, year	Search databases (time)	Quality for primary studies	No. of studies (major design)	Participants/ Cases	Health outcome	Exposure (comparison)	ES (95%CI)
						Fall vs. non-fall	0.978（0.966-0.990）
						Winter vs. non-winter	1.038 (1.019–1.057)
						Month of birth:	RR
						Jan. vs. non-Jan.	**1.055 (1.033–1.077)**
						Feb. vs. non-Feb.	1.007 (0.963–1.052)
						Mar. vs. non-Mar.	1.019 (0.979–1.060)
						Apr. vs. non-Apr.	0.992 (0.965–1.020)
						May vs. non-May	0.994 (0.973–1.016)
						Jun. vs. non-Jun.	0.973 (0.945–1.002)
						Jul. vs. non-Jul.	0.987 (0.965–1.010)
						Aug. vs. non-Aug.	**0.965 (0.941–0.991)**
						Sep. vs. non-Sep.	**0.967 (0.942–0.993)**
						Oct. vs. non-Oct.	**0.981 (0.969–0.994)**
						Nov. vs. non-Nov.	0.987 (0.965–1.010)
						Dec. vs. non-Dec.	**1.046 (1.014–1.078)**
				>29074024/ 118397	Bipolar I disorder*	season of birth:	RR
						Spring vs. non-spring	0.993 (0.970–1.016)
						Summer vs. non-summer	0.986 (0.968–1.004)
						Fall vs. non-fall	1.000 (0.978–1.023)
						Winter vs. non-winter	**1.020 (1.001–1.041)**
						Month of birth:	RR
						Jan. vs. non-Jan.	**1.039 (0.990–1.090)**
						Feb. vs. non-Feb.	1.013 (0.986–1.041)
						Mar. vs. non-Mar.	1.031 (0.976–1.089)
						Apr. vs. non-Apr.	0.972 (0.946–1.000)
						May vs. non-May	0.983 (0.940–1.027)
						Jun. vs. non-Jun.	0.990 (0.961–1.019)

Author, year	Search databases (time)	Quality for primary studies	No. of studies (major design)	Participants/ Cases	Health outcome	Exposure (comparison)	ES (95%CI)
						Jul. vs. non-Jul.	0.983 (0.957–1.010)
						Aug. vs. non-Aug.	0.968 (0.919–1.019)
						Sep. vs. non-Sep.	0.977 (0.937–1.019)
						Oct. vs. non-Oct.	1.007 (0.981–1.033)
						Nov. vs. non-Nov.	1.022 (0.982–1.063)
						Dec. vs. non-Dec.	**1.026 (0.977–1.077)**
				>29074024/ 751702	Major depressive disorder	season of birth:	RR
						Spring vs. non-spring	0.994 (0.980–1.009)
						Summer vs. non-summer	0.993 (0.978–1.009)
						Fall vs. non-fall	1.005 (0.993–1.016)
						Winter vs. non-winter	**1.010 (1.004–1.017)**
						Month of birth:	RR
						Jan. vs. non-Jan.	1.006 (0.995–1.017)
						Feb. vs. non-Feb.	**1.014 (1.004–1.025)**
						Mar. vs. non-Mar.	1.015 (0.984–1.047)
						Apr. vs. non-Apr.	0.980 (0.959–1.001)
						May vs. non-May	0.996 (0.965–1.027)
						Jun. vs. non-Jun.	0.984 (0.952–1.017)
						Jul. vs. non-Jul.	1.000 (0.972–1.030)
						Aug. vs. non-Aug.	1.004 (0.987–1.021)
						Sep. vs. non-Sep.	1.008 (0.993–1.024)
						Oct. vs. non-Oct.	1.005 (0.986–1.025)
						Nov. vs. non-Nov.	0.996 (0.966–1.027)
						Dec. vs. non-Dec.	1.010 (1.000–1.021)
				>29074024/ 148793	Alcohol use disorder	season of birth:	RR
						Spring vs. non-spring	**0.980 (0.961–0.999)**
						Summer vs. non-summer	1.009 (0.985–1.032)

Author, year	Search databases (time)	Quality for primary studies	No. of studies (major design)	Participants/ Cases	Health outcome	Exposure (comparison)	ES (95%CI)
						Fall vs. non-fall	**1.016 (1.003–1.030)**
						Winter vs. non-winter	0.996 (0.983–1.010)
						Month of birth:	RR
						Jan. vs. non-Jan.	0.995 (0.973–1.018)
						Feb. vs. non-Feb.	0.996 (0.972–1.021)
						Mar. vs. non-Mar.	0.993 (0.969–1.017)
						Apr. vs. non-Apr.	0.973 (0.914–1.034)
						May vs. non-May	0.989 (0.924–1.057)
						Jun. vs. non-Jun.	0.984 (0.959–1.009)
						Jul. vs. non-Jul.	1.001 (0.977–1.026)
						Aug. vs. non-Aug.	1.027 (0.977–1.080)
						Sep. vs. non-Sep.	1.012 (0.989–1.036)
						Oct. vs. non-Oct.	1.013 (0.991–1.036)
						Nov. vs. non-Nov.	**1.023 (1.00002–1.047)**
						Dec. vs. non-Dec.	0.998 (0.974–1.021)
				>29074024/ 118743	Alzheimer's disease	season of birth:	RR
						Spring vs. non-spring	0.985 (0.961–1.010)
						Summer vs. non-summer	0.978 (0.954–1.002)
						Fall vs. non-fall	1.012 (0.993–1.031)
						Winter vs. non-winter	1.023 (0.995–1.051)
						Month of birth:	RR
						Jan. vs. non-Jan.	1.042 (0.991–1.095)
						Feb. vs. non-Feb.	1.039 (0.975–1.107)
						Mar. vs. non-Mar.	0.987 (0.961–1.013)
						Apr. vs. non-Apr.	0.994 (0.922–1.072)
						May vs. non-May	0.973 (0.909–1.042)
						Jun. vs. non-Jun.	0.969 (0.917–1.023)

Author, year	Search databases (time)	Quality for primary studies	No. of studies (major design)	Participants/ Cases	Health outcome	Exposure (comparison)	ES (95%CI)
						Jul. vs. non-Jul.	0.994 (0.954–1.037)
						Aug. vs. non-Aug.	0.965 (0.920–1.014)
						Sep. vs. non-Sep.	**1.038 (1.001–1.075)**
						Oct. vs. non-Oct.	0.998 (0.972–1.025)
						Nov. vs. non-Nov.	0.999 (0.972–1.026)
						Dec. vs. non-Dec.	0.988 (0.962–1.015)
				>29074024/6615	anorexia nervosa	season of birth:	RR
						Spring vs. non-spring	0.987 (0.926–1.052)
						Summer vs. non-summer	0.981 (0.920–1.046)
						Fall vs. non-fall	1.022 (0.960–1.088)
						Winter vs. non-winter	1.012 (0.950–1.078)
						Month of birth:	RR
						Jan. vs. non-Jan.	1.016 (0.909–1.135)
						Feb. vs. non-Feb.	1.011 (0.885–1.156)
						Mar. vs. non-Mar.	0.943 (0.845–1.053)
						Apr. vs. non-Apr.	0.988 (0.883–1.106)
						May vs. non-May	1.031 (0.924–1.150)
						Jun. vs. non-Jun.	1.002 (0.896–1.120)
						Jul. vs. non-Jul.	0.939 (0.839–1.051)
						Aug. vs. non-Aug.	1.003 (0.899–1.118)
						Sep. vs. non-Sep.	0.995 (0.892–1.109)
						Oct. vs. non-Oct.	1.027 (0.909–1.161)
						Nov. vs. non-Nov.	1.042 (0.934–1.163)
						Dec. vs. non-Dec.	1.002 (0.897–1.118)
				>29074024/10563	bulimia nervosa	season of birth:	RR
						Spring vs. non-spring	0.954 (0.904–1.006)
						Summer vs. non-summer	1.036 (0.983–1.091)

Author, year	Search databases (time)	Quality for primary studies	No. of studies (major design)	Participants/ Cases	Health outcome	Exposure (comparison)	ES (95%CI)
						Fall vs. non-fall	1.023 (0.973–1.075)
						Winter vs. non-winter	0.988 (0.938–1.040)
						Month of birth:	RR
						Jan. vs. non-Jan.	0.969 (0.884–1.063)
						Feb. vs. non-Feb.	1.002 (0.919–1.092)
						Mar. vs. non-Mar.	0.957 (0.874–1.047)
						Apr. vs. non-Apr.	0.937 (0.853–1.030)
						May vs. non-May	0.967 (0.882–1.061)
						Jun. vs. non-Jun.	1.046 (0.954–1.148)
						Jul. vs. non-Jul.	1.009 (0.921–1.106)
						Aug. vs. non-Aug.	1.052 (0.964–1.148)
						Sep. vs. non-Sep.	1.040 (0.953–1.135)
						Oct. vs. non-Oct.	1.045 (0.960–1.137)
						Nov. vs. non-Nov.	0.982 (0.899–1.073)
						Dec. vs. non-Dec.	0.990 (0.906–1.082)
Pantavou et al., 2020	Pubmed and Scopus (August 2018)	NR(NR)	22(Case-control studies)	75315222/ 145672	multiple sclerosis	season of birth:	OR
						Spring vs. Fall	**1.14 (1.04, 1.24)**
						Summer vs. Fall	**1.08 (1.00, 1.17)**
						Winter vs. Fall	1.07 (0.99,1.15)
						Month of birth:	OR
						Jan. vs. Oct.	1.01(0.97,1.06)
						Feb. vs. Oct.	1.02(0.97,1.06)
						Mar. vs. Oct.	**1.05 (1.00, 1.11)**
						Apr. vs. Oct.	**1.12 (1.05, 1.21)**
						May vs. Oct.	**1.07 (1.00, 1.14)**
						Jun. vs. Oct.	1,02(0,98,1.05)
						Jul. vs. Oct.	1.02(0.98,1.05)

Author, year	Search databases (time)	Quality for primary studies	No. of studies (major design)	Participants/ Cases	Health outcome	Exposure (comparison)	ES (95%CI)
						Aug. vs. Oct.	1.03(0.99,1.08)
						Sep. vs. Oct.	1.05(0.99,1.11)
						Nov. vs. Oct.	**0.96 (0.93, 0.99)**
						Dec. vs. Oct.	1.00(0.96,1.06)
Susanto et al., 2017	PubMed, Web of Science, Scopus, Google Scholar and ProQuest Health(NR)	>70% high quality (Cochrane)	10(Cohort studies)	119979/ 1834	cord blood IgE	season of birth	OR
						Spring vs. Summer	1.05(0.81,1.35)
						Fall vs. Summer	1.17(0.92,1.48)
						Winter vs. Summer	**1.24(1.01,1.52)**
Karalexi et al., 2020	unpublished primary data(NR)	NR(NR)	10(cohort studies)	18211601/ 4987	Total CNS tumors	season of birth	IRR (Total vs. Boys vs. Girls)
						Spring vs. non-spring	1.04 (0.98–1.11) vs. 1.09 (0.96–1.23) vs. 1.03 (0.92–1.14)
						Summer vs. non-summer	0.94 (0.87–1.01) vs. 0.93 (0.85–1.02) vs. 0.96 (0.83–1.10)
						Fall vs. non-fall	0.98 (0.90–1.07) vs. 0.94 (0.85–1.03) vs. 0.94 (0.85–1.05)
						Winter vs. non-winter	1.06 (0.99–1.14) vs. 1.07 (0.98–1.18) vs. 1.08 (0.98–1.20)
				18211601/ 1507	Astrocytomas	Spring vs. non-spring	1.06 (0.95–1.19) vs. 0.95 (0.75–1.21) vs. 1.23 (1.03–1.46)
						Summer vs. non-summer	1.03 (0.92–1.16) vs. 1.07 (0.90–1.26) vs. 1.04 (0.86–1.25)
						Fall vs. non-fall	0.94 (0.78–1.14) vs. 0.92 (0.77–1.10) vs. 0.88 (0.66–1.18)
						Winter vs. non-winter	1.03 (0.90–1.17) vs. 1.08 (0.91–1.28) vs. 1.10 (0.82–1.46)
				18211601/ 1463	Embryonal tumors	Spring vs. non-spring	0.99 (0.88–1.12) vs. 1.00 (0.84–1.19) vs. 1.00 (0.81–1.24)
						Summer vs. non-summer	0.90 (0.80–1.02) vs. 0.89 (0.75–1.06) vs. 0.94 (0.76–1.16)
						Fall vs. non-fall	1.00 (0.89–1.13) vs. 0.96 (0.77–1.19) vs. 1.08 (0.88–1.33)

Author, year	Search databases (time)	Quality for primary studies	No. of studies (major design)	Participants/ Cases	Health outcome	Exposure (comparison)	ES (95%CI)
						Winter vs. non-winter	**1.13 (1.01–1.27)** vs. 1.24 (1.05–1.46) vs. 1.04 (0.84–1.28)
Tung et al., 2022	PubMed, Cochrane Library and EMBASE(October 20, 2021)	5 high-quality research(NOS)	5(Cohort studies)	103986/ 12343	Postpartum depression	season of childbirth	RR
						Spring vs. Winter	0.96 (0.92–1.00)
						Summer vs. Winter	0.88 (0.73–1.05)
						Fall vs. Winter	**0.86 (0.75–0.99)**
						non-winter vs. Winter	**0.83 (0.78–0.88)**
Liao et al., 2023	Cochrane Library, PubMed, EMBASE, and Web of Science(September 29, 2021)	8 high-quality research(NOS)	11(Cohort studies)	2,816,293/ 87,466	Hypertensive Disorders in Pregnancy	season of childbirth	OR
						Spring vs.Summer	1.09(0.97-1.22）
						Fall vs.Summer	1.01(0.92-1.11）
						Winter vs.Summer	**1.18(1.02-1.38）**
Hsu et al., 2022*	PubMed, Embase, and Cochrane （September 30,2021）	No high bias research (Hoy's risk of bias tool)	8 （Observational studies）	>29074024/3776	narcolepsy	season of birth:	RR(Overall vs. Asia vs. North America vs. Europe)
						Spring vs. non-spring	1.04 (0.96–1.12) vs.0.95 (0.85–1.06) vs.**1.21 (1.06–1.38)** vs.1.05 (0.92–1.19)
						Summer vs. non-summer	1.01 (0.93–1.09) vs.0.95 (0.85–1.06) vs.1.02 (0.89–1.17) vs.1.06 (0.92–1.21)
						Fall vs. non-fall	0.98 (0.91–1.06) vs.1.09 (0.99–1.21) vs.0.87 (0.74–1.01) vs.0.91 (0.79–1.05)
						Winter vs. non-winter	1.02 (0.94–1.10) vs.1.04 (0.94–1.16) vs.0.97 (0.84–1.13) vs.1.01 (0.89–1.16)
						Month of birth:	RR(Overall vs. Asia vs. North America vs. Europe)
						Jan. vs. non-Jan.	1.06 (0.94–1.20) vs.1.05 (0.89–1.23) vs.0.88 (0.67–1.15) vs.1.14 (0.91–1.43)
						Feb. vs. non-Feb.	0.96 (0.84–1.10) vs.0.99 (0.84–1.17) vs.0.95 (0.73–1.24) vs.0.93 (0.73–1.18)
						Mar. vs. non-Mar.	1.11 (0.99–1.26) vs.0.91 (0.77–1.08) vs.**1.47 (1.20–1.79)** vs.1.13 (0.91–1.40)
						Apr. vs. non-Apr.	0.90 (0.78–1.03) vs.**0.80 (0.66–0.97)** vs.0.96 (0.74–1.25) vs.0.97 (0.77–1.23)

Author, year	Search databases (time)	Quality for primary studies	No. of studies (major design)	Participants/ Cases	Health outcome	Exposure (comparison)	ES (95%CI)
						May vs. non-May	1.09 (0.96–1.24) vs.1.13 (0.95–1.33) vs.1.10 (0.87–1.39) vs.1.04 (0.83–1.30)
						Jun. vs. non-Jun.	0.99 (0.87–1.13) vs.0.95 (0.79–1.13) vs.0.95 (0.74–1.23) vs.1.04 (0.82–1.31)
						Jul. vs. non-Jul.	0.91 (0.80–1.04) vs.0.83 (0.69–1.00) vs.1.09 (0.87–1.37) vs.0.90 (0.70–1.14)
						Aug. vs. non-Aug.	1.11 (0.98–1.26) vs.1.07 (0.91–1.25) vs.1.00 (0.79–1.27) vs.1.22 (0.98–1.52)
						Sep. vs. non-Sep.	0.92 (0.81–1.06) vs.1.09 (0.92–1.28) vs.**0.75 (0.56–0.99)** vs.0.82 (0.64–1.06)
						Oct. vs. non-Oct.	1.01 (0.89–1.15) vs.1.04 (0.89–1.22) vs.0.91 (0.70–1.18) vs.1.03 (0.81–1.30)
						Nov. vs. non-Nov.	1.02 (0.89–1.16) vs.1.15 (0.99–1.34) vs.0.93 (0.71–1.21) vs.0.88 (0.68–1.13)
						Dec. vs. non-Dec.	1.03 (0.90–1.17) vs.1.08 (0.92–1.26) vs.1.08 (0.85–1.37) vs.0.96 (0.75–1.22)
Córdova-Palomera et al., 2015*	PubMed,PsycINFO, and Web of Science(October 22, 2013)	4 high-quality research (STROBE)	7(observational studies)	29676/ 30102	subclinical psychosis	Season of birth:	OR（Adult vs. Children)
						Winter vs. non-winter	1.22(0.87,1.70) vs.**1.12(1.03,1.21)**

CI - confidence interval; IRR - incidence rate ratio; NH - Northern Hemisphere; NOS - Newcastle-Ottawa Scale; NR - not reported; OR - odds ratio; RR - risk ratio; SH - Southern Hemisphere; STROBE - Strengthening the Reporting of Observational Studies in Epidemiology.

Bold effect estimates indicate statistically significant associations.

Hemisphere-specific estimates or definitions were retained when available; otherwise, pooled estimates were used.

Winter/summer definitions: NH = Dec-Feb/Jun-Aug; SH = Jun-Aug/Dec-Feb.

*Included in the general analysis but excluded from the final evidence-grading analysis (Table 2) because of insufficient data: fewer than three studies in significant subgroups or incomplete reporting of included studies.

Table S3. Excluded studies and reasons for exclusion after full-text review

ID	Title	Reason for exclusion
1	Association between relative age at school and persistence of ADHD in prospective studies: an individual participant data meta-analysis	No data for specific months
2	Associations of Meteorology with Adverse Pregnancy Outcomes: A Systematic Review of Preeclampsia, Preterm Birth and Birth Weight	No forest plot for sub-studies
3	Association between maternal depression and maternal sensitivity from birth to 12 months: a meta-analysis	Analyzes only absolute age without considering the specific birth month or season
4	Pregnancy in Systemic Sclerosis: Results of a Systematic Review and Meta-analysis	Focuses only on birth weight
5	Female Reproductive Performance and Maternal Birth Month: A Comprehensive Meta-Analysis Exploring Multiple Seasonal Mechanisms	Discusses birth temperature and rainfall without specific months
9	A systematic review and meta-analysis of Northern Hemisphere season of birth studies in schizophrenia	Missing values in the forest plot
10	Season of birth and anorexia nervosa	No forest plot for sub-studies
11	Timing of neonatal mortality and severe morbidity during the postnatal period: a systematic review	Review without meta-analysis
12	Confounding in association studies: month of birth and multiple sclerosis	Review without meta-analysis
13	Geographical and seasonal correlation of multiple sclerosis to sporadic schizophrenia	Review without meta-analysis
14	Season of birth and primary central nervous system tumors: a systematic review of the literature with critical appraisal of underlying mechanisms	No meta-analysis
15	A systematic review on the association of month and season of birth with future anthropometric measures	Review without meta-analysis
18	Perinatal Risk Factors for Asthma and Allergic Rhinitis in Children and Adolescents	No meta-information for birth month
20	Analysis of DNA methylation at birth and in childhood reveals changes associated with season of birth and latitude	Is an epigenome-wide meta-analysis study
21	Short-term ambient temperature variations and incidence of preterm birth: A systematic review and meta-analysis	Focuses on environmental temperature rather than specific months
22	A systematic review and meta-analysis of heat exposure impacts on maternal fetal and neonatal health	Involves heat exposure with no analysis of months or seasons
23	Perinatal light imprinting of circadian clocks and systems (PLICCS): A signature of photoperiod around birth on circadian system stability and association with cancer	Review without meta-analysis
24	Perinatal photoperiod associations with bipolar disorder and depression: A systematic literature review and cross-sectional analysis of the UK Biobank database	Review without meta-analysis
25	The Association Between Season and Hypertensive Disorders in Pregnancy: a Systematic Review and Meta-analysis	Forest plots not included
26	Food allergy is linked to season of birth, sun exposure, and vitamin D deficiency	Only a systematic review without meta-analysis
27	Season of birth and schizophrenia: a systematic review and meta-analysis of data from the Southern Hemisphere	The article is older, and the forest plot lacks numbers
28	Effects of temperature, weather, seasons, atmosphere, and climate on the exacerbation of inflammatory bowel diseases: A systematic review and meta-analysis	Studies general climate levels, not birth-related factors
30	Seasonality of month of birth in patients with autoimmune endocrine diseases: A systematic review	Review without meta-analysis
31	Association between a large change between the minimum and maximum monthly values of solar insolation and a history of suicide attempts in bipolar I disorder	Focuses only on birth location
32	The impact of relative age effects on psychosocial development: A systematic review	Only relative age without specific birth month or season
33	The role of latitude and infections in the month-of-birth effect linked to schizophrenia	No forest plot
34	Attention Deficit Hyperactivity Disorder and Relative Age: A Meta-Analysis	Relative age cannot calculate the season

ID	Title	Reason for exclusion
35	Prevalence and risk factors of respiratory syncytial virus in children under 5 years of age in the WHO European Region: A systematic review and meta-analysis	No meta-analysis on months and seasons
36	The prevalence of long-term neurodevelopmental outcomes in preterm-born children in low- and middle-income countries: a systematic review and meta-analysis of developmental outcomes in 72 974 preterm-born children	No meta-analysis on the birth month and season of premature infants
39	Month of birth as a risk factor for multiple sclerosis: an update	Review without meta-analysis
41	Do prenatal factors shape the risk for dementia?: A systematic review of the epidemiological evidence for the prenatal origins of dementia	Review without meta-analysis
42	Prevalence of Babies Born With Neural Tube Defects and Geospatial Mapping of Therapeutic Services: A Systematic Review	Review without meta-analysis

Table S4. AMSTAR 2 assessment of included meta-analyses

Study	Q1	Q2	Q3	Q4	Q5	Q6	Q7	Q8	Q9	Q10	Q11	Q12	Q13	Q14	Q15	Q16	Overall
Córdova-Palomera et al., 2015*	Y	N	N	PY	Y	N	N	PY	Y	N	Y	Y	Y	Y	Y	Y	CL
Susanto et al., 2017	Y	N	N	PY	Y	Y	N	PY	PY	N	Y	Y	Y	Y	Y	Y	CL
Pantavou et al., 2020	Y	N	N	PY	Y	Y	N	PY	N	N	Y	N	Y	Y	Y	Y	CL
Karalexi et al., 2020	Y	N	N	N	N	N	PY	PY	N	N	Y	Y	Y	Y	Y	Y	CL
Calov et al., 2020	Y	N	N	PY	Y	Y	N	PY	PY	N	N	Y	Y	N	Y	Y	CL
Hsu et al., 2021	Y	PY	PY	PY	Y	PY	Y	Y	Y	N	Y	Y	Y	Y	Y	Y	L
Tung et al., 2022	Y	N	N	PY	Y	Y	N	PY	Y	N	Y	Y	Y	Y	Y	Y	CL
Liao et al., 2023	Y	PY	N	PY	Y	PY	N	PY	Y	N	Y	Y	Y	Y	Y	Y	L
Hsu et al., 2022*	Y	PY	N	PY	Y	PY	Y	Y	Y	N	Y	Y	Y	Y	Y	Y	CL
Coury et al., 2023*	Y	PY	N	PY	Y	Y	N	PY	PY	N	Y	Y	Y	Y	Y	Y	CL

AMSTAR 2 - A Measurement Tool to Assess Systematic Reviews 2; CL - critically low; H - high; L - low; M - moderate; N - no; NMA - no meta-analysis conducted; PY - partial yes; Q - question; Y - yes.

*Included in the general analysis but excluded from the final evidence-grading analysis because of insufficient data.
